# Subsidence and carbon dioxide emissions in a smallholder peatland mosaic in Sumatra, Indonesia

**DOI:** 10.1007/s11027-018-9803-2

**Published:** 2018-03-21

**Authors:** Ni’matul Khasanah, Meine van Noordwijk

**Affiliations:** 1 Southeast Asia Regional Programme, World Agroforestry Centre (ICRAF), Jl. CIFOR, Situgede, Sindang Barang, Bogor, 16115 Indonesia; 2 Plant Production Systems, Department of Plant Sciences, Wageningen University and Research, 6708 PB Wageningen, the Netherlands

**Keywords:** Agroforestry, CO_2_ emissions, Fertilizer application, Peat subsidence, Smallholder, Tropical peatlands

## Abstract

Most attention in quantifying carbon dioxide (CO_2_) emissions from tropical peatlands has been on large-scale plantations (industrial timber, oil palm (*Elaeis guinensis*)), differing in drainage and land-use practices from those of smallholder farms. We measured subsidence and changes in bulk density and carbon organic content to calculate CO_2_ emissions over 2.5 years in a remnant logged-over forest and four dominant smallholder land-use types in Tanjung Jabung Barat District, Jambi Province, Sumatra, Indonesia: (1) simple rubber (*Hevea brasiliensis*) agroforest (> 30 years), (2) mixed coconut (*Cocos nucifera*) and coffee gardens (*Coffea liberica*) (> 40 years), (3) mixed betel nut (*Areca catechu*) and coffee gardens (> 20 years), and (4) oil palm plantation (1 year). We quantified changes in microtopography for each site for greater accuracy of subsidence estimates and tested the effects of nitrogen and phosphorus application. All sites had a fibric type of peat with depths of 50 to > 100 cm. A recently established oil palm had the highest rate of peat subsidence and emission (4.7 cm year^−1^ or 121 Mg CO_2_ ha^−1^ year^−1^) while the remnant forest had the lowest (1.8 cm year^−1^ or 40 Mg CO_2_ ha^−1^ year^−1^). Other land-use types subsided by 2–3 cm year^−1^, emitting 70–85 Mg CO_2_ ha^−1^ year^−1^. Fertilizer application did not have a consistent effect on inferred emissions. Additional emissions in the first years after drainage, despite groundwater tables of 40 cm, were of the order of belowground biomass of peat forest. Despite maintaining higher water tables, smallholder landscapes have CO_2_ emissions close to, but above, current IPCC defaults.

## Introduction

Indonesia has experienced the world’s highest land-based carbon (C) emissions over the past decades owing to a combination of forest conversion (Margono et al. 2014), peatland drainage (Tata et al. 2014; Thorburn and Kull 2015), and land-clearing fires that escaped control (Turetsky et al. 2015). Indonesia has also, however, been an early champion of climate-change mitigation measures in the forest and peatland sectors (van Noordwijk et al. 2014a; Busch et al. 2015) and of an integrated policy environment for combining adaptation and mitigation aspects from local to national levels (Agung et al. 2014; Di Gregorio et al. 2017). In developing land-use policies, rather than separate policies for forestry and agriculture, the specific issue of tropical peatlands and the fires and haze caused by their conversion has played an important role (Abood et al. 2015, Wijedasa et al. 2017, Larsen et al. 2018). Current scenario models (Mulia et al. 2014; Suwarno et al. 2018a, b) are, however, constrained by a lack of reliable data for emissions from existing smallholder land-use systems on peat.

Page et al. (2011b) estimated that 56% (24.8 Mha) of the global area of tropical peatlands is in Southeast Asia, mostly in Indonesia (20.6 Mha) and Malaysia (2.5 Mha). While recent estimates of peat areas in Africa and Latin America have increased (Gumbricht et al. 2017), most of the current carbon dioxide (CO_2_) emissions from tropical peatland occur in Southeast Asia owing to high forest conversion rates. Approximately 35% of the Indonesian peatland area (7.2 Mha) is in Sumatra (Wahyunto et al. 2003), with other areas mainly in Kalimantan and Papua. As long as other land was available for conversion, peat swamps were mostly bypassed by development, with smallholder mosaic agriculture nibbling at the edges. Large-scale conversion started in Indonesia and Malaysia in the 1990s, when conflicts over land tenure in other forest areas could be avoided by shifting to the peat-covered parts of the landscape. Large areas have been drained for agricultural use, mostly oil palm (*Elaeis guinensis*) and pulpwood plantations (Miettinen et al. 2016), producing continuous CO_2_ emissions, subsidence, and changes to the peat’s characteristics owing to drainage.

To prevent subsidence and emissions, groundwater levels should be maintained between 40 cm below and 100 cm above the peat surface. This recommendation by Wösten et al. (2008) has been used as a generic policy standard in Indonesia. The rate of CO_2_ emissions of large-scale plantations has been widely studied (Wakhid et al. 2017; Sumarga et al. 2016; Carlson et al. 2015; Page et al. 2011a), but little is yet known of the subsidence and emission dynamics in the specific context of smallholder mosaic landscapes. Nonetheless, mandated groundwater levels for rewetted peat landscapes are applied to smallholder landscapes as well as plantations. Technical drainage specifications are based on avoiding crop damage in the wettest places (typically in between drainage canals), with a management trade-off between the distance of canals (and thus total length of canals) and the water table to be maintained in the canals (van Noordwijk et al. 2014b). Smallholder peatland mosaics have made different choices in this trade-off compared to large-scale operators with more technical means to make deeper canals further apart. In the current debate, opportunities for low-drainage, low-carbon-emission peatland livelihoods are highly sought after but have hardly been evaluated.

Tropical peats are mostly water. With 5–15% dry matter content, they are essentially a suspended litter layer of dead leaves, branches, and occasional tree trunks arrested in early stages of decomposition, where structural coherence is primarily obtained from tree roots (Page et al. 1999). As anyone who has walked in a tropical peat swamp knows, beyond the roots one can sink deeply, before finding a branch or trunk that holds. Carbon accumulation in tropical peat, compared to other forests, occurs not because of high plant production but rather because of slow decomposition of roots and wood under anaerobic conditions (Chimner and Ewel 2005). Southeast Asian peat swamps can contain up to 10,000 years of litter accumulation in peat domes more than 10 m thick at their core. The carbon storage per meter of peat depends primarily on the bulk density, ranging from 250 to 750 Mg ha^−1^ of C, which exceeds the aboveground C storage of tropical rainforests, accumulated at a rate of 0.5–1 mm year^−1^ or 0.25–5 Mg C ha^−1^ year^−1^ (Tiemeyer and Kahle, 2014; Kurnianto et al. 2015). When such peatlands are drained, the initial rate of subsidence is several centimeters per year owing to a combination of consolidation (increase in bulk density) and decomposition (releasing the net accumulation of 30–100 years (Wösten et al. 1997; Hooijer et al. 2010; Hooijer et al. 2012)). The ratio between consolidation and decomposition tends to decrease with time (Frolking et al. 2010). Subsequent decomposition can both increase and decrease bulk density, in the absence of weight that leads to compaction (Hooijer et al. 2012). Aerobic microflora is responsible for the increase in decomposition rate after drainage (Nurulita 2016), initially with little help from the litter organisms that comminute litter in aerobic forest soils (Garcia-Palacios et al. 2016). The microflora may well be nutrient (nitrogen (N), phosphorus (P)) limited, as peat swamp-forests function at high C:N and C:P ratios. Some published evidence exists for N and P effects on temperate zone and tropical peat decomposition (Crill et al. 1994; Jauhiainen et al. 2014; Song et al. 2013; Reeza et al. 2014). Handayani (2009) documented an initial response of respiration after N addition to peat soils from Aceh (Sumatra). Maswar (2011) in a study of subsidence and emissions in recently opened peat swamps under various types of land use at the same site found emissions in the first 3–5 years after drainage to be substantially higher than in the subsequent period. The literature is clear on the decline over time of subsidence and decomposition rates but not on the process-level explanation (van Noordwijk et al. 2014b). As decomposing bacteria themselves do not keep track of time, explanations could be based on a changing quality of remaining substrate (once the more easily decomposable pools have been exhausted), the circumstances (return to wetter conditions after subsidence and structural collapse), or a combination of both. The total additional emissions in early years in the Maswar (2011) data amount to a pool size of 100–200 Mg C ha^−1^, similar to the belowground biomass of the forest that preceded it.

The rate of peatland CO_2_ emissions is large but so is the uncertainty of available estimates. Measured rates of CO_2_ emissions from drained peatlands vary widely, with depth of water table, climate, peat temperature (Marwanto and Agus 2014), and farming practices recognized as sources of variation (Hooijer et al. 2012; Carlson et al. 2015; Maswar 2011). Variation in the fraction of fresh wood debris in the peat, according to Paramananthan (2010a, b) and Veloo et al. (2015), may well have to be added to the commonly used fibric/hemic/sapric classification of “peat maturity” and stage of decomposition. Bulk density and ash content are partially correlated with peat maturity. Existing published estimates, derived with some variation in methods, range widely: 20 Mg CO_2_ ha^−1^ year^−1^ (Carlson et al. 2015), 2.4–48 Mg CO_2_ ha^−1^ year^−1^ (Maswar 2011), 44.0–58.7 Mg CO_2_ ha^−1^ year^−1^ (DID and LAWOO 1996), 58.4–74.5 Mg CO_2_ ha^−1^ year^−1^ (Couwenberg and Hooijer 2013), and 72.7 (Othman et al. 2011) to 100 Mg CO_2_ ha^−1^ year^−1^ (Hooijer et al. 2012). Part of this variation may reflect genuine differences in local contexts, but variations in methods and associated biases cannot be excluded. While chamber-based estimates (Wakhid et al. 2017) require scaling up from measurement periods to an annual basis and face challenges in the day-night rhythms of respiration and in separating root from peat-based respiration as described by Marwanto and Agus (2014), the subsidence measurements suffer from uncertainties in the dynamics of microtopography of the peat surface as common measurement protocols for subsidence suggest a single reading of height relative to a rod that is fixed below the peat layers (Couwenberg and Hooijer 2013).

**Fig. 1:**
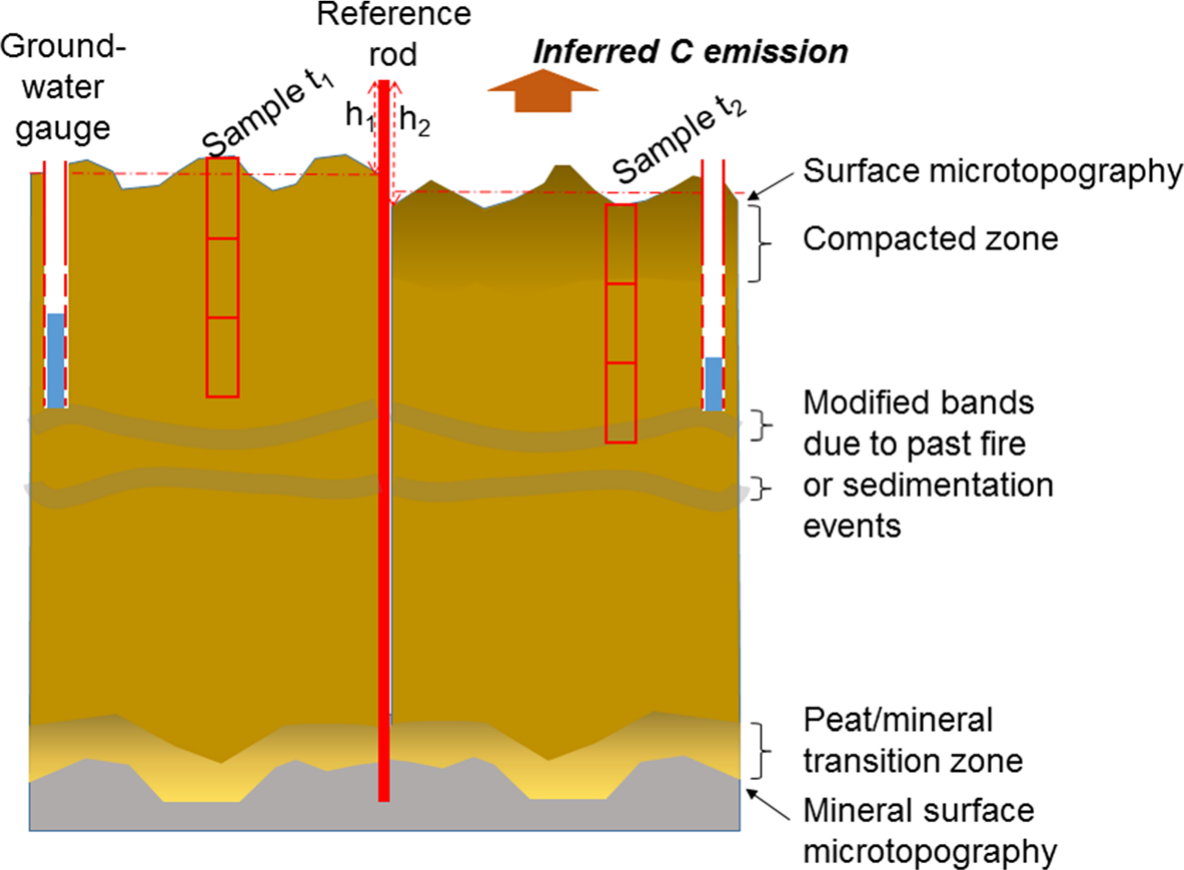
Schematic representation of the challenge to infer C emissions from measured height change with at least two-time intervals (here mirrored around the rod) in the face of compaction, dynamics of surface microtopography, and presence of bands of modified peat from past disturbances

The underlying mineral soil as well as the surface have more relief than in standard diagrammatic representations (Fig. 1), and spatial variation in peat depth at the scale of annual subsidence rates is considerable. Dynamics of microtopography around the measurement point may thus be confounded with overall subsidence (Maswar 2011). Rather than using a single-depth measurement, local mapping of topography around the measurement points might give more certain results. Page et al. (2009) commented that “at the local scale the peat surface microtopography of hummocks, comprising tree bases, and hollows, which are interspersed with tree breathing roots, reduce the water flow rate and help maintain the water table close to or above the surface throughout the year.” It is not clear at what temporal scale this microtopography is changing. Beyond variation in water-table depth throughout the year, differences in nutrient supply might also influence results with the specific effects of fertilizer application largely untested. An alternative method for estimating cumulative CO_2_ emissions since the start of drainage is based on the assumption that ash components are conservative and that increasing ash concentration indicates C loss (Grønlund et al. 2008; Maswar 2011). This method relies on estimates of pre-drainage ash content, for example, derived from the ash content in deeper layers of the same profile. The advantage of this method is that single point measurements suffice but it has not been adequately compared with data from actual change monitoring.

Of the total area of Tanjung Jabung Barat District, Jambi Province, Sumatra, Indonesia, approximately 40% (200,000 ha) is peatland (Wahyunto et al. 2003) and 8% (16,065 ha) of that is *hutan lindung gambut* (HLG) or peat protection forest. In the 1970s, over-exploitation of logging concessions converted primary peat forest to logged-over forest (Widayati et al. 2012), which was later claimed by smallholders, drained, and cultivated with coconut (*Cocos nucifera*), rubber (*Hevea brasiliensis*), and coffee (*Coffea liberica*) systems. Recently, large-scale plantations of oil palm and fast-growing pulpwood (*Acacia mangium* and *Acacia crassicarpa*) were established. Conflicts over the land rights assigned to them by the central government became violent (Galudra et al. 2014). CO_2_ emissions from drained peat are a major issue in the area. The objective of this study was to estimate CO_2_ emissions (Mg CO_2_ ha^−1^ year^−1^) of different land-use types. We quantified peat subsidence and characteristics under smallholder management in relation to the length of time after drainage and fertilizer application. Specific questions for the measurement and data analysis of smallholder land-use systems on peat were fourfold.

1. Is there any variation of subsidence and emissions between land-use types and time after drainage (earlier compared to recent drainage)?
2. Does the average of multiple readings of subsidence by taking into account dynamics of microtopography reduce uncertainty relative to a single reading of subsidence?
3. Do changes in ash content reflect the rate of emissions?
4. Does fertilization (nitrogen, phosphorus) affect subsidence and/or emissions?

## Methods

### Study site

The study was conducted in Tanjung Jabung Barat District on the east coast of Jambi Province, Sumatra, Indonesia. Conditions here represented the eastern coastal peat swamp zone of Sumatra, which constitutes roughly one third of the peat area in Indonesia. It is one of the peat areas most intensively used for smallholder land-use systems. Based on the Köppen climate classification, the study area is classified as A_f_ with minimum, mean, and maximum annual air temperatures of 21, 30, and 32 °C, respectively, and a mean annual rainfall of 2324–2373 mm year^−1^. During the study period, November 2012–May 2015, rainfall in 2013 was above average (3208 mm year^−1^) (Badan Pusat Statistik Kabupaten Tanjung Jabung Barat 2014).

### Measurement locations and experimental design

**Fig. 2:**
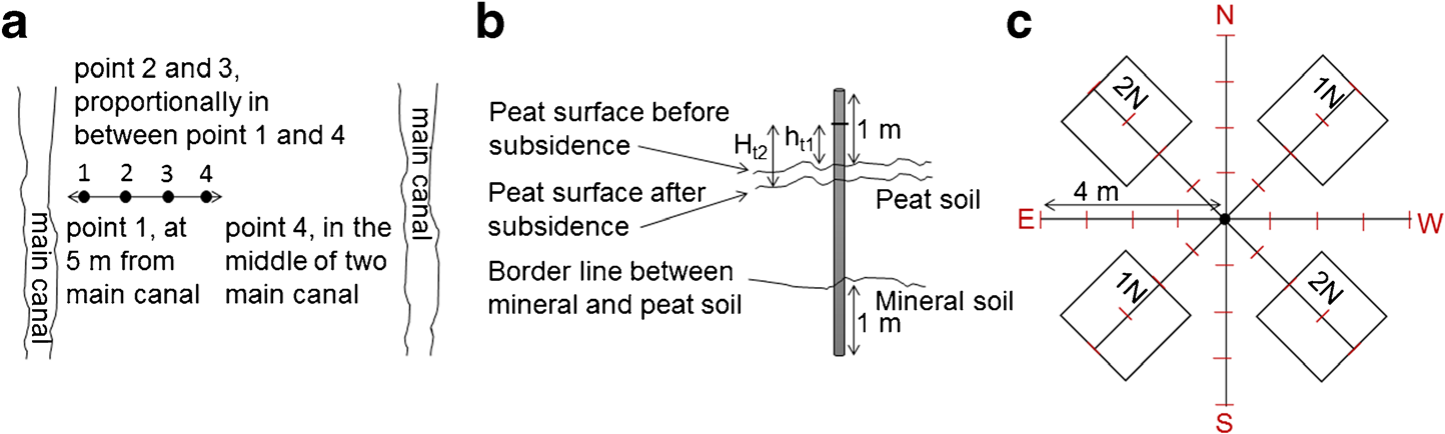
Design of peat subsidence measurement. Four positions of metal rods in transects perpendicular to the main drainage canal to measure peat subsidence (**a**); illustration of peat subsidence measurement at each metal rod (**b**); and design of fertilizer application treatment (1N and 2N refer to the amount of fertilizer in Table 1) (**c**)

We measured the rate of CO_2_ emissions based on measurement of peat subsidence and analysis of peat characteristics in four dominant land-use types managed by smallholders in the region with two to three replications for each land-use type: (1) simple rubber (*Hevea brasiliensis*) agroforest, (2) mixed coconut (*Cocos nucifera*) and coffee (*Coffea liberica*), (3) mixed betel nut (*Areca catechu*) and coffee, and (4) oil palm (*Elaeis guinensis*) plantation. The period after drainage varied 20–40 years (> 20 years for mixed betel nut and coffee, > 30 years for simple rubber agroforest, and > 40 years for mixed coconut and coffee), except for oil-palm plantation, at 1 year after drainage, but it had been previously logged many years ago. All sites had fibric peat with depths of 50–> 100 cm. The four dominant land-use types reflected different stages in the local land-use change trajectory. We could not apply a full factorial design of land-use types and time after drainage, specifically, smallholder oil palm could only be sampled in the early stages of its life cycle. As a reference, we also measured the rate of CO_2_ emissions in logged-over forest with natural drainage rather than canals. As the peat thickness and the depth of the water table of drained peatland vary, depending on distance to drainage canals (Hooijer et al. 2012; Maswar 2011), in each replication, we used four measurement points (Fig. 2a) in transects perpendicular to the main drainage canal, covering a wide range of peat thicknesses and depths of the water table.

To test the local effects of increased nitrogen and phosphorus nutrition on peat decomposition and subsidence under the prevailing water management regime, we designed the fertilizer application treatment with three levels (0N, 1N, and 2N) based on the doses recommended for oil palm (Table 1), following a six-monthly schedule. As illustrated in Fig. 2c, fertilizer subplots were 2 × 2 m^2^, with subsidence measurements focused on their center. Fertilizer application was tested in three different land-use types: (1) simple rubber agroforest, (2) oil-palm plantation, and (3) logged-over forest, and at measurement points 1 and 4 in Fig. 2a, to test contrasting conditions of peat and water-table depth.

**Table 1:** Doses of fertilizer (N and P) application for each treatment and age of palm per measurement point and application

Treatment	Age of palm (years)	No. of applications	Urea	TSP	Urea	TSP
			(kg/tree/application)^1)^		(kg/m^2^/application)	
0N	–	–	–	–	–	–
1N	1	2	0.63	0.63	0.28	0.28
	2	2	0.75	0.75	0.33	0.33
	3	2	0.75	0.75	0.33	0.33
2N	1	2	1.25	1.25	0.55	0.55
	2	2	1.50	1.50	0.66	0.66
	3	2	1.50	1.50	0.66	0.66

^1^Assuming the rates are applied over 2.27 m^2^ (1.7-m-radius circle around the tree). Source: Mutert et al. (1999)

### Peat subsidence measurements

In November 2012 at each replication at each measurement point (Fig. 2a), the monitoring of peat subsidence began with the installing of metal rods. At each measurement point, a permanent mark (h_t1_) was made on the metal rod to indicate the initial point of measurement. Peat subsidence (h_t2_… h_tn_) was monitored every 6 months for 2.5 years (November 2012–May 2015) (Fig. 2b). To quantify heterogeneity of subsidence and dynamics of microtopography around the metal rods, relative heights in eight cardinal directions were also mapped surrounding the central points of the metal rods, at 2–4-m length with 10-cm intervals (Fig. 2c). For the 0N fertilizer application treatment, we used 484 microtopography points in each replication of oil palm, logged-over forest, and rubber and 324 microtopography points in each replication of mixed betel nut and coffee and mixed coconut and coffee. For the 1N and 2N fertilizer application treatments, we used 82 microtopography points in each replication and treatment. For each measurement point, the rate of subsidence (cm year^−1^) was then calculated separately, with negative subsidence accepted for points that appeared to rise.

### Peat-characteristics analysis

Every 6 months at each measurement point and fertilizer application treatment, peat samples to 30-cm depth at 10-cm intervals were taken 0.5–1 m from the metal rod using the Eijkelkamp peat auger (the sample was easily contained in the auger) following Agus et al. (2011). During measurement of microtopography, site compaction by access to the plot could not be fully avoided. The peat samples were taken to the laboratory for bulk density, ash, and organic C content analysis. Bulk density was analyzed by drying the sample at 105 °C for 48 h or until the sample reached stable dry weight; ash and organic C content were analyzed based on the loss on ignition (LOI) method (Agus et al. 2011).

### Water-table measurement

The depth of the water table at 2 m away from the subsidence measurement point was monitored every month using perforated PVC tubes. At each replication, the depth of the water table was calculated over four different measurement points and dates.

### Estimation of the rate of CO_2_ emission

The rate of CO_2_ emission was then estimated from surface height loss (subsidence) and the characteristic of peat (bulk density and C organic content) after the period of loss (Eq. 1). The assumption is, after the end of the consolidation phase that follows immediately after drainage, compaction and oxidation are the only causes of surface height loss.

1 $$ C={S}_t\times {BD}_t\times {C}_t\times 3.67 $$

where *C* is annual CO_2_ emissions (Mg CO_2_ ha^−1^ year^−1^), *S*_*t*_ is the annual surface height loss (cm year^−1^), *C*_*t*_ is the organic C content (%) after the loss, *BD*_*t*_ is bulk density (g cm^−3^) after the loss, and 3.67 is a conversion from C to CO_2_. The relative weight loss on ignition is the complement of the relative ash content provided the organic matter content, with estimates of the C concentration in organic matter (which depends essentially on the C:O ratio of the latter) derived from literature.

We also compared the rate of emission based on subsidence to ash content differences (before and after the period of loss), modified from Grønlund et al. (2008), which can be used to estimate cumulative emissions since drainage based on a single measurement:

2 $$ C=\left(\left(\left({A}_2\times {BD}_2\times T\right)\times \left(\raisebox{1ex}{$1$}\!\left/ \!\raisebox{-1ex}{${A}_1$}\right.-1\right)-\left(\left(1-{A}_2\right)\times {BD}_2\times T\right)\right)/1.922\right)\times 100\times 3.67/t $$

where *A*_2_ is ash concentration measured after *t* years of change, *A*_1_ is (inferred) the ash concentration before the loss, *BD*_2_ is bulk density (g cm^−3^) at the measurement time, *T* is the thickness of the soil sample, and 1.922, 3.67, and 100 are conversion factors from the mass of soil to C, from C to CO_2_, and from grams per square centimeter to megagrams per hectare, respectively.

A similar calculation (Eq. 1 and Eq. 2) was also applied to estimate the rate of CO_2_ emissions because of fertilizer application.

### Statistical data analysis

Characteristics of peat (bulk density, ash and organic C content) were analyzed for the effect of the single factor of date of measurement, leaving replication, fertilizer application, depth of sampling, and measurement points as co-variates, using SYSTAT 11. In the statistical analysis, a 5% probability of type I errors was accepted in rejecting null hypotheses of no difference.

## Results

### Dynamics of microtopography

**Fig. 3:**
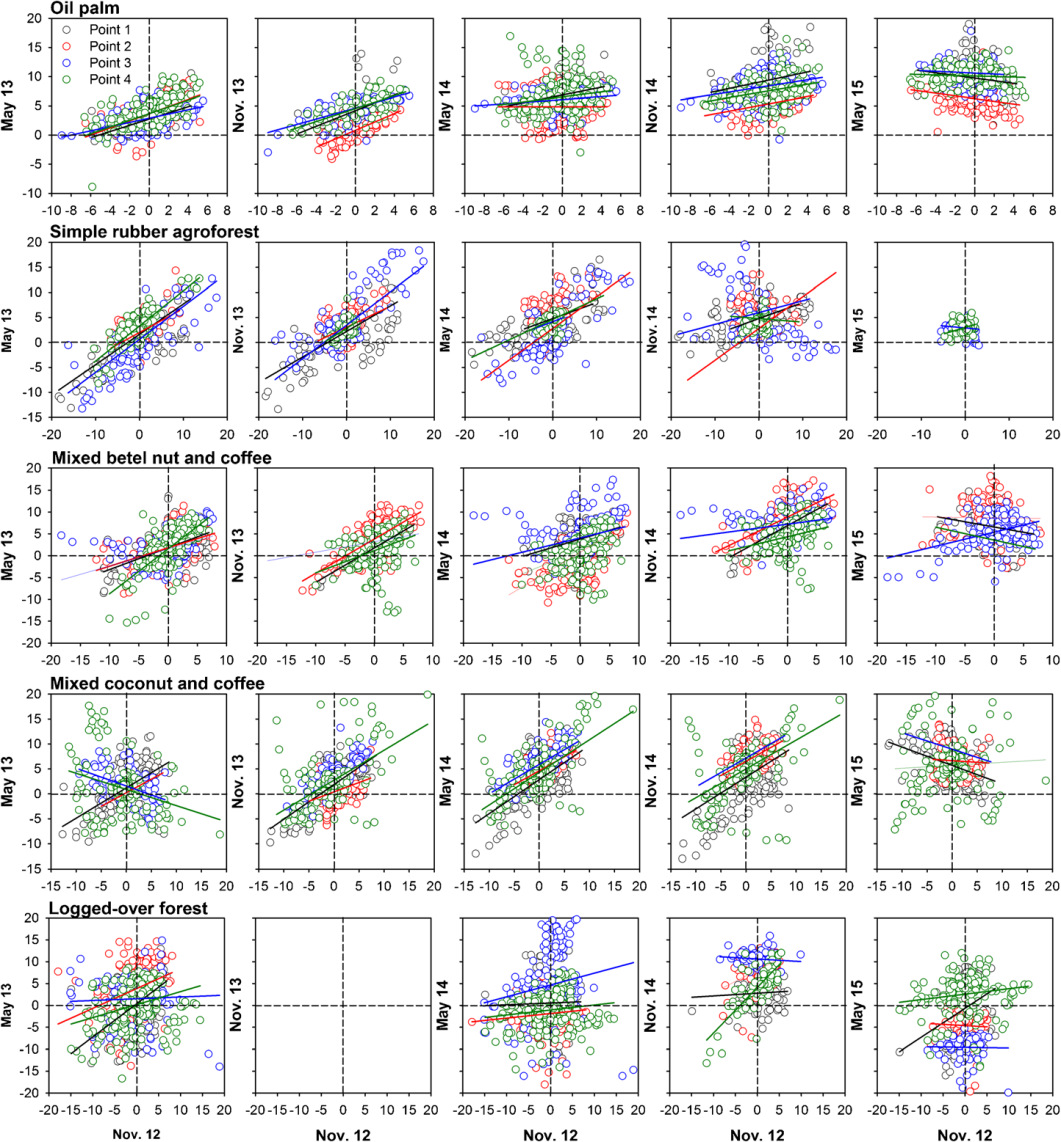
Deviation (difference between values at certain points of measurement and average value at first measurement) of microtopography levels of different land-use types, distance to canal, and time of measurement. *X* axis is deviation at *t* and *Y* axis is deviation at *t* + 1

**Fig. 4:**
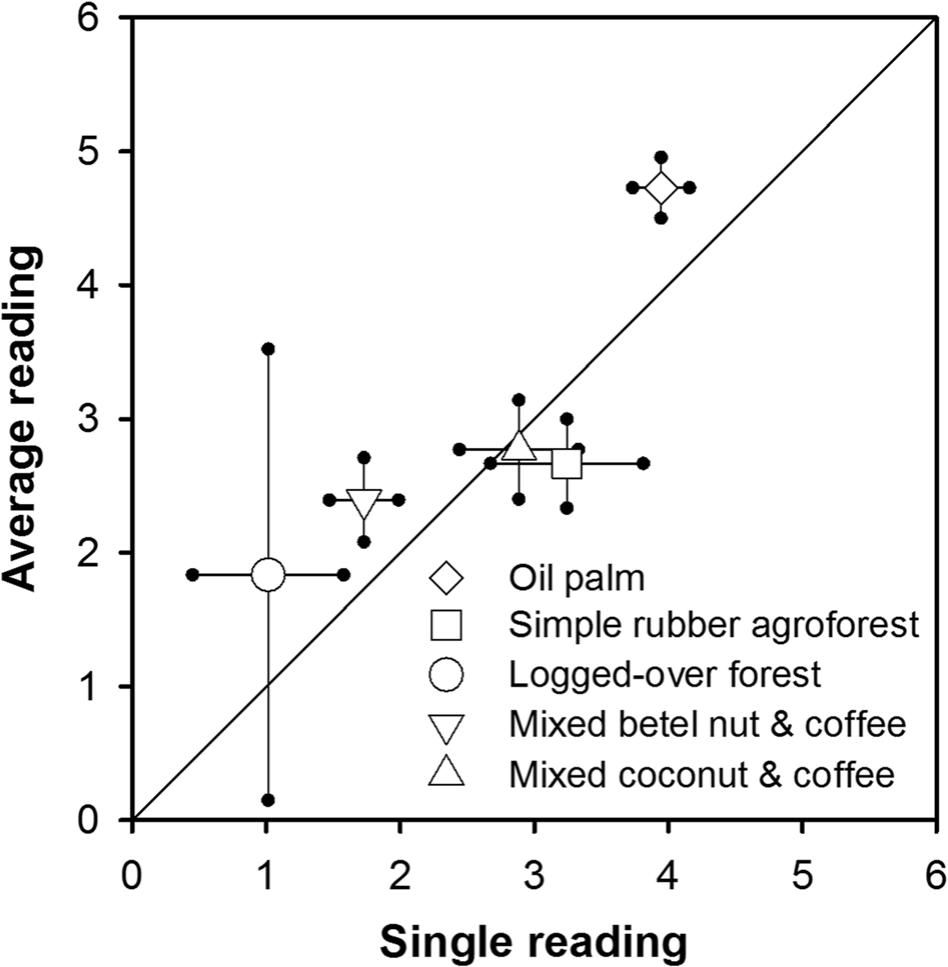
The inferred rate of peat subsidence (cm year^−1^) based on a single reading at the central metal rod compared to the average of multiple readings for all microtopography sites

Figure 3 presents deviation (the difference between the value at a certain point of measurement and the average value at first measurement) of microtopography levels of different land-use types, distance to canal, and time of measurement. In general, it shows that in all measured land-use types and distance to canals, the deviation has shifting trends from time to time. It indicates that the level of subsidence is not homogenous over the soil surface. Homogenous subsidence occurs if the trend has 45° of slope. Further analyses of confidence intervals of single and multiple readings (Fig. 4) found that the confidence interval of multiple readings is not always narrower than that of a single reading. The rate of subsidence based on multiple readings was slightly higher than that based on a single reading.

### Peat characteristics

**Fig. 5:**
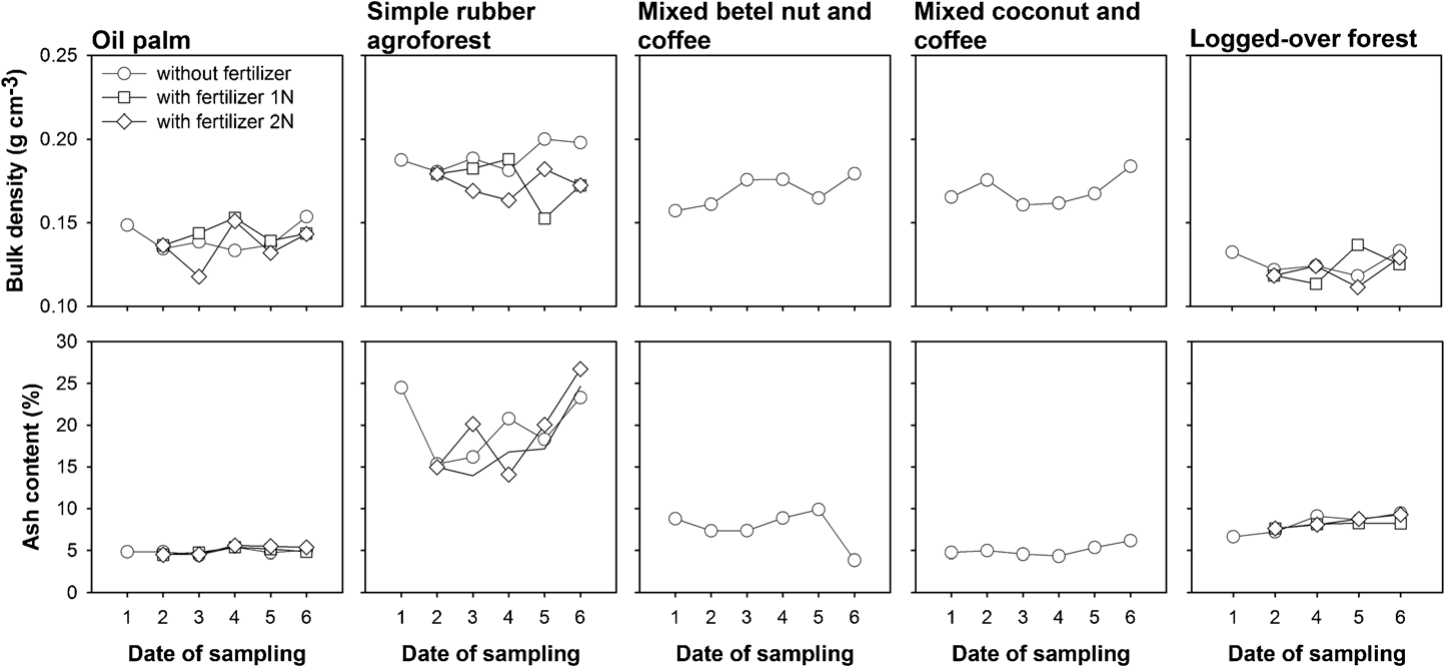
Mean of bulk density (g cm^−3^) and ash content (%) for different land-use types. Date of sampling: 1, Nov 2012; 2, May 2013; 3, Nov 2013; 4, May 2014; 5, Nov 2014; 6, May 2015

The bulk density and ash content of different land-use types are presented in Fig. 5. The bulk density and ash content are the average of replication and distance to the canal. Overall, the ash content and bulk density of each land-use type did not show differences (*p* < 0.05) among distance to the canal and fertilizer application, except for the ash content in oil palm (*Elaeis guinensis*) and simple rubber (*Hevea brasiliensis*) agroforest (differences among distances to the canal) and bulk density in a simple rubber agroforest (differences among fertilizer applications). In terms of the date of sampling, the ash content of each land-use type tended to increase by time and show differences (*p* < 0.05) among dates of measurement. By contrast, the bulk density of each land-use type did not show differences (*p* < 0.05) among dates of measurement, except for oil palm. Oil palm was the only site examined that was 1 year after drainage. Among the land-use types, the highest and the lowest bulk densities were found in the simple rubber agroforest and logged-over forest, respectively, while the highest and lowest ash content were found in the simple rubber agroforest and oil palm, respectively.

### Peat subsidence and emissions

The pattern of peat characteristics that show differences among dates of measurement (Fig. 5) but not among distances to the canal allowed us to estimate the rates of emission (Mg CO_2_ ha^−1^ year^−1^) of different land-use types and fertilizer applications based on peat subsidence and bulk density and organic C content of each measurement date. However, unclear patterns of emissions led us to use the average value of bulk density and organic C content over all dates of measurement and present the rates as the weighted average of distance to the canal.

#### Emissions based on peat subsidence and peat characteristics compared to ash content differences

**Fig. 6:**
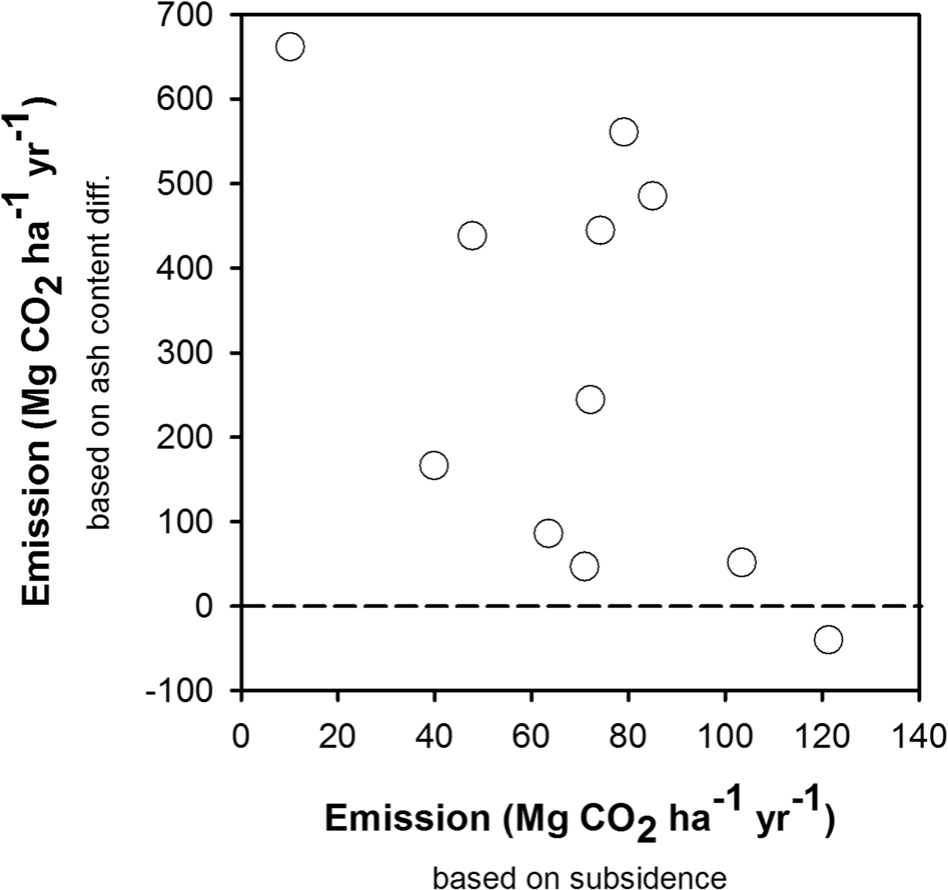
Emissions (Mg CO_2_ ha^−1^ year^−1^) based on subsidence and peat characteristics (*C*_org_ and bulk density) compared to ash content differences

Figure 6 presents the comparison of the rate of emission based on subsidence and peat characteristics (Eq. 1) with the rate of emission based on ash content differences (Eq. 2). The latter provided extremely high emission estimates negatively correlated with results of subsidence measurement. For subsequent analysis, we relied on the subsidence measurements.

#### Emissions of different fertilizer applications and land-use types

Table 2 shows that fertilizer application did not have a consistent effect on the rates of peat subsidence and emission. The highest rates of peat subsidence and emission were found in the recently established oil palm (4.7 cm year^−1^ or 121 Mg CO_2_ ha^−1^ year^−1^) and the lowest in the reference plot with natural canals and logged-over forest (0.5 cm year^−1^ or 10.2 Mg CO_2_ ha^−1^ year^−1^). Other land-use types that had drained more than 20 years had 2–3 cm year^−1^ of subsidence or 70–85 Mg CO_2_ ha^−1^ year^−1^.

**Table 2:** Average of peat subsidence rate (cm year^−1^), bulk density (g cm^−3^), *C*_org_ (%), and peat emission rate (Mg CO_2_ ha^−1^ year^−1^) of different land-use types and fertilizer applications

Land-use types	Fertilizer applications	Years after drainage	Subsidence rate (cm year^−1^)	Bulk density (g cm^−3^)	*C*_org_ (%)	Emissions (Mg CO_2_ ha^−1^ year^−1^)
Oil palm	0	1	4.7	0.14	49.4	121.4
	1		4.2	0.14	49.3	103.5
	2		2.6	0.13	49.3	63.5
Simple rubber agroforest	0	> 30	2.7	0.20	41.4	79.1
	1		2.7	0.18	42.4	74.2
	2		2.5	0.18	42.9	72.2
Logged-over forest	0	–	1.8	0.12	47.6	39.9
	1		2.2	0.12	47.7	47.8
	2		0.5	0.11	47.5	10.2
Mixed betel nut and coffee	0	> 20	2.4	0.17	48.1	71.0
Mixed coconut and coffee	0	> 40	2.8	0.17	49.4	85.0

**Fig. 7:**
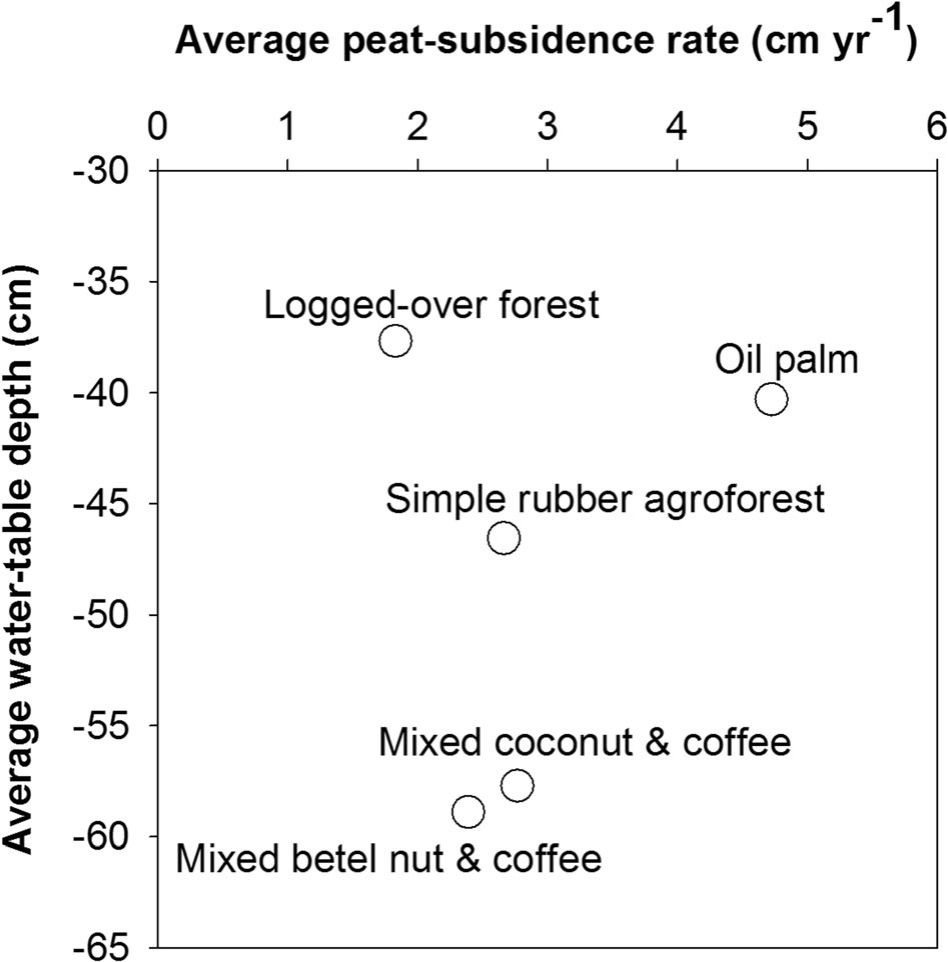
Average of water-table depth (cm) and peat-subsidence rate (Mg CO_2_ ha^−1^ year^−1^) of different land uses at no fertilizer application

Further analysis by plotting the average of the water-table depth and the rate of subsidence showed that the deeper the water table, the higher the rate of subsidence (Fig. 7), but this only occurred at sites drained more than 20 years ago (simple rubber agroforest, mixed coconut and coffee, and mixed betel nut and coffee) or the reference site with natural canals and logged-over forest. At the recently established site with oil palm, although the water-table depth was less than those sites with longer periods after drainage, the rate of subsidence was high.

## Discussion

We estimated the annual rates of peat subsidence (cm year^−1^) and CO_2_ emissions (Mg CO_2_ ha^−1^ year^−1^) of different land-use types under smallholder management. Most studies quantifying CO_2_ emissions from tropical peatlands have been focused on large-scale plantations of commodities, such as oil palm (*Elaeis guinensis*) and pulpwood (Jauhiainen et al. 2012; Hooijer et al. 2012; Page et al. 2011a). The CO_2_ emissions from smallholder peat land-use systems with less intensive drainage systems have not received enough attention. The study was designed to answer four questions. In response to the first question regarding variation of subsidence and emission between land-use types and time after drainage (earlier compared to recent drainage), our study confirmed that early stages of drainage lead to rapid collapse, even with fairly high groundwater tables. The recently established oil palm subsided 4.7 cm year^−1^, emitting 121 Mg CO_2_ ha^−1^ year^−1^. However, this value is significantly lower than what was reported in a review on peat CO_2_ emissions from oil palm and pulpwood large plantations. Peat emissions in the early stages of plantation drainage are about 178 Mg CO_2_ ha^−1^ year^−1^ (Page et al. 2011a). Other land-use types more than 20 years after drainage subsided by 2–3 cm year^−1^, emitting 70–85 Mg CO_2_ ha^−1^ year^−1^. This value is slightly lower than what was reported in the same review (86 Mg CO^2−^ eq ha^−1^ year^−1^, annualized over 50 years).

Our estimates were higher than the recent peat-oxidation emission values for tropical peatland set by the Intergovernmental Panel on Climate Change (IPCC 2014), which suggested default values of 51 Mg CO_2_ ha^−1^ year^−1^ for smallholder systems, 55 Mg CO_2_ ha^−1^ year^−1^ for commercial plantations (oil palm, industrial timber), and 10 Mg CO_2_ ha^−1^ year^−1^ for disturbed secondary forest. Estimates by Miettinen et al. (2017) of cumulative carbon emissions since 1990 from peat oxidation in Peninsular Malaysia, Sumatra and Borneo, based on the IPCC defaults, may thus be on the low side. Their estimate that 34% of emissions so far have occurred in smallholder areas and 44% in industrial plantations (mostly oil palm and industrial timber), and the remaining 22% from disturbed forests, would not be much different if our results were added to the emission-factor database, as emission factors would increase for all land uses. Ishikura et al. (2018) reported subsidence of 1.55–1.62 cm year^−1^ for oil palm in Sarawak (Malaysia), with corresponding CO_2_ emissions, measured in chambers and after correction for root respiration, of around 40 Mg CO_2_ ha^−1^ year^−1^. A further analysis of the differences in substrate (peat type) and details of groundwater dynamics will be needed to reduce uncertainty in the estimates.

In relation to the second question, several studies on microtopography of peatland reported that the formation of the microtopography of the peat surface is a product of an interaction of autogenic and allogenic processes (Nungesser 2003), with others noting the effects of water-table fluctuation, tree diversity (Lampela et al. 2016; Shi et al. 2015), and wildfire (Benscoter et al. 2005; Benscoter et al. 2015), though those processes might be random (Lampela et al. 2016). Our subsidence measurement confirmed that the level of subsidence over the soil surface was heterogenous, and consequently, multiple readings, by considering the microtopographical dynamics of subsidence, would be more accurate.

Regarding the third question (use of the Grønlund et al. (2008) equation), the results in Fig. 6 showed that for our 0.5–1-m peat depth setting, estimation of emissions based on one-off (without subsidence recording) measurement of peat characteristics (bulk density and organic C content), with inferred ash content differences to a pre-drainage control, provided high and unstable values compared to the commonly used subsidence method. Although the method may give some early indications in a soil-survey context, close scrutiny of the validity of the underlying assumptions would be needed before it could be used in confidence. Warren et al. (2012) found within a specific data set that the relationship between bulk density and ash content was sufficiently tight to estimate the second from the first. Farmer et al. (2014) found this assumption to be unreliable where multiple land-use histories were involved. The dynamic of C organic content and approximately stable bulk density indicated that the main contribution of peat subsidence is oxidation, or the decomposition process, and a small effect of compaction during measurement, though the plots had been drained 20–40 years ago. In line with this result, Hooijer et al. (2012) reported that oxidation or decomposition was not only the main contribution of peat subsidence at the early stage of peat drainage but can also contribute at the later stages.

For the last question, emission effects of fertilizer application on peat subsidence were small relative to effects of water-table depth. This result is in line with findings reported by Oktarita et al. (2017), where the impact of fertilizer-induced emissions was minimal, though under fully controlled experimental conditions, fertilizer application has been shown to increase the decomposition rate (Reeza et al. 2014). The response of tree-root systems to local nutrient enrichment may contribute to differences between field results and those obtained in conditions where microbial processes dominate.

Overall, our data with long-term emission rates for smallholder land uses in the range of 70–85 Mg CO_2_ ha^−1^ year^−1^, along with the spatial analysis by Miettinen et al. (2017), support specific attention to emissions from peatland under smallholder management. As shown by Miettinen et al. (2017) and Warren et al. (2017), emissions during land-clearing fires, which have received considerable public attention, are less than half of the total emissions caused by disturbing and converting the remaining peat forests. From a global emissions perspective, the recurrent emissions need to be controlled, with the fine-tuning of water management in already converted peat landscapes a high priority. Current water management depends primarily on uncontrolled drainage in open canals and affects adjacent forests, shifting them from carbon sinks to carbon sources (Miettinen et al. 2017). Effective solutions will require peat hydrological units to be reconciled with the scale at which land-use decisions are made in practice (Ritzema et al. 2014; Evers et al. 2017; Suwarno et al. 2018b).

## Conclusion

Our research found that emission estimates based on peat subsidence can be improved by taking microtopography into account, using multiple readings around measurement rods. The partial independence of local surface dynamics relates to the dynamics of water-table depth; root activity and accumulation of litter on the soil surface may need to be included in estimates of the rate of peat CO_2_ emissions of drained peatlands. The rate of peat CO_2_ emissions based on the subsidence rate between two different measuring times in combination with peat characteristics (bulk density and C organic content) provided a better estimation than an ash-based “internal tracer” method. Long-term drainage can be expected to decrease the rate of CO_2_ emissions at a given groundwater depth, with additional emissions in the early stages of the same order as decayed root biomass of the preceding vegetation, while fertilizer application did not show a strong effect on the rates of peat subsidence and emissions.
